# Comment on “Facial Atrophy in Oral Submucous Fibrosis: An Association or a Coincidence”

**DOI:** 10.1155/2016/5747458

**Published:** 2016-11-07

**Authors:** Sonal Grover, George Koshy

**Affiliations:** Department of Oral & Maxillofacial Pathology & Microbiology, Christian Dental College, CMC, Ludhiana, Punjab, India

With reference to a case report titled “Facial Atrophy in Oral Submucous Fibrosis: An Association or a Coincidence” [1] published in your esteemed journal, we would like to emphasize the importance of considering systemic sclerosis (SS) as an important differential diagnosis in oral submucous fibrosis (OSMF).

Systemic sclerosis is a clinically heterogeneous generalized disorder which affects the connective tissue of the skin and internal organs such as gastrointestinal tract, lungs, heart, and kidneys. It is characterized by enormous deposition of collagen, alterations of the microvasculature, and disturbances of the immune system [2]. Oral manifestations in SS are very common and these include limited ability to open the mouth, facial and tongue rigidity, thinning of lips, xerostomia, periodontal disease, increased periodontal ligament width, and osseous resorption of the mandible [2, 3]. A few decades back, plenty of cases have been reported with neurological manifestations as well [4, 5].

Oral submucous fibrosis (OSMF) is a chronic insidious collagen related disorder associated with betel quid chewing and characterized by progressive hyalinization of the submucosa. The hallmark of the disease is submucosal fibrosis that affects most parts of the oral cavity, causing progressive trismus with rigid lips, cheeks, pharynx, and upper third of the esophagus leading to dysphagia [6]. Since extensive fibrosis plays a key role in the pathogenesis of OSMF also, it is not possible to distinguish OSMF from SS histologically.

In the OSMF case reported [1], the patient had extensive atrophy of facial muscle along with restricted mouth opening. The striking finding in this case was cerebral and cerebellum atrophy. This finding should have prompted the authors to rule out the possibility of SS, as extensive facial atrophy with neurological manifestations is more likely to be seen in SS than in OSMF. Except for the epithelial dysplasia observed, all the remaining findings are common to SS as well. The epithelial dysplasia can easily be correlated with the long term consumption of areca nut by the patient. The diagnostic criteria for SS are as given below:

*Major Criterion*

- Proximal diffuse (truncal) sclerosis (skin tightness, thickening, and nonpitting induration)

*Minor Criteria*

- Sclerodactyly (only fingers and/or toes)
- Digital pitting scars or loss of substance of the digital finger pads (pulp loss)
- Bibasilar pulmonary fibrosis

The patient should fulfill the major criterion or two of the three minor criteria.

Thus, through this letter, the authors intend to highlight the differential diagnosis of SS in OSMF cases involving parts of the body other than the oral cavity and pharynx.
